# The role of apolipoprotein E (*APOE*) genotype in early mild cognitive impairment (E-MCI)

**DOI:** 10.3389/fnagi.2013.00011

**Published:** 2013-04-01

**Authors:** Shannon L. Risacher, Sungeun Kim, Li Shen, Kwangsik Nho, Tatiana Foroud, Robert C. Green, Ronald C. Petersen, Clifford R. Jack, Paul S. Aisen, Robert A. Koeppe, William J. Jagust, Leslie M. Shaw, John Q. Trojanowski, Michael W. Weiner, Andrew J. Saykin, for the Alzheimer's Disease Neuroimaging Initiative (ADNI)

**Affiliations:** ^1^Department of Radiology and Imaging Sciences, Center for Neuroimaging, Indiana University School of MedicineIndianapolis, IN, USA; ^2^Center for Computational Biology and Bioinformatics, Indiana University School of MedicineIndianapolis, IN, USA; ^3^Department of Medical and Molecular Genetics, Indiana University School of MedicineIndianapolis, IN, USA; ^4^Division of Genetics, Department of Medicine, Brigham and Women's Hospital and Harvard Medical SchoolBoston, MA, USA; ^5^Department of Neurology, Mayo ClinicRochester, MN, USA; ^6^Department of Radiology, Mayo ClinicRochester, MN, USA; ^7^Department of Neurology, University of CaliforniaSan Diego, San Diego, CA, USA; ^8^Department of Radiology, University of MichiganAnn Arbor, MI, USA; ^9^Department of Neurology, University of CaliforniaBerkeley, Berkeley, CA, USA; ^10^Department of Pathology and Laboratory Medicine, University of Pennsylvania School of MedicinePhiladelphia, PA, USA; ^11^Departments of Radiology, Medicine, and Psychiatry, University of CaliforniaSan Francisco, San Francisco, CA, USA; ^12^Department of Veterans Affairs Medical CenterSan Francisco, CA, USA; ^†^Data used in preparation of this article were obtained from the Alzheimer's Disease Neuroimaging Initiative (ADNI) database (adni.loni.ucla.edu). As such, the investigators within the ADNI contributed to the design and implementation of ADNI and/or provided data but did not participate in analysis or writing of this report. A complete listing of ADNI investigators can be found at: http://adni.loni.ucla.edu/wp-content/uploads/how_to_apply/ADNI_Acknowledgement_List.pdf

**Keywords:** apolipoprotein E (APOE), early mild cognitive impairment (E-MCI), Florbetapir/AV-45/Amyvid, positron emission tomography (PET), magnetic resonance imaging (MRI), cerebrospinal fluid (CSF), Alzheimer's disease neuroimaging initiative (ADNI)

## Abstract

**Objective:** Our goal was to evaluate the association of *APOE* with amyloid deposition, cerebrospinal fluid levels (CSF) of Aβ, tau, and p-tau, brain atrophy, cognition and cognitive complaints in E-MCI patients and cognitively healthy older adults (HC) in the ADNI-2 cohort.

**Methods:** Two-hundred and nine E-MCI and 123 HC participants from the ADNI-2 cohort were included. We evaluated the impact of diagnostic status (E-MCI vs. HC) and *APOE* ε4 status (ε4 positive vs. ε4 negative) on cortical amyloid deposition (AV-45/Florbetapir SUVR PET scans), brain atrophy (structural MRI scans processed using voxel-based morphometry and Freesurfer version 5.1), CSF levels of Aβ, tau, and p-tau, and cognitive performance and complaints.

**Results:** E-MCI participants showed significantly impaired cognition, higher levels of cognitive complaints, greater levels of tau and p-tau, and subcortical and cortical atrophy relative to HC participants (*p* < 0.05). Cortical amyloid deposition and CSF levels of Aβ were significantly associated with *APOE* ε4 status but not E-MCI diagnosis, with ε4 positive participants showing more amyloid deposition and lower levels of CSF Aβ than ε4 negative participants. Other effects of *APOE* ε4 status on cognition and CSF tau levels were also observed.

**Conclusions:** *APOE* ε4 status is associated with amyloid accumulation and lower CSF Aβ, as well as increased CSF tau levels in early prodromal stages of AD (E-MCI) and HC. Alternatively, neurodegeneration, cognitive impairment, and increased complaints are primarily associated with a diagnosis of E-MCI. These findings underscore the importance of considering *APOE* genotype when evaluating biomarkers in early stages of disease.

## Introduction

Alzheimer's disease (AD) is the most common age-related neurodegenerative disease, featuring cognitive decline, accumulation of amyloid plaques and neurofibrillary tangles, and extensive neurodegeneration (Alzheimer's Association, 2011; McKhann et al., 2011). The most commonly accepted prodromal AD stage is mild cognitive impairment (MCI), which is characterized by clinically-relevant cognitive dysfunction in the absence of significant interference with daily functioning (Petersen et al., 1999; Albert et al., 2011). Amnestic MCI features marked memory impairments which are predictive of progression to clinical AD. Recently, MCI patients have been classified into two forms based on severity: early MCI (E-MCI) and late MCI (L-MCI). Relative to an age-appropriate normative level, E-MCI patients show an approximately 1–1.5 standard deviation (SD) decline in memory, while L-MCI patients show a 1.5 SD or greater decline. These designated cut-offs for E-MCI and L-MCI have not been fully explored to date. However, the identification of participants with a 1–1.5 SD deficit in memory as E-MCI may be more sensitive for identifying participants in the earliest stages of cognitive decline. However, the specificity of these diagnostic criteria has yet to be determined and may be lower than the L-MCI cut-offs, allowing participants with more diverse causal factors of cognitive decline (other than prodromal AD) to be included in this diagnostic category. Future studies examining these clinical criteria and clinical and pathological outcomes of identified E-MCI patients relative to L-MCI patients will be important for understanding the cognitive changes observed in these patients. Importantly, these new guidelines provide an opportunity to evaluate the role of AD biomarkers and other potential disease-causing factors in a very early clinical stage. In fact, a recent study demonstrated increased amyloid binding measured using [^18^F]Florbetapir positron emission tomography (PET) in patients with E-MCI relative to HC, but no alterations in metabolism as assessed using [^18^F]FDG PET (Wu et al., 2012).

The most common genetic variant associated with late-onset AD is the apolipoprotein E (*APOE*) ε4 allele (Corder et al., 1993; Bertram et al., 2010). The presence of an ε4 allele confers a significantly higher likelihood of developing AD. *APOE* genotype is also associated with AD biomarkers, with the presence of an *APOE* ε4 allele associated with greater amyloid deposition (Drzezga et al., 2009; Morris et al., 2010; Fleisher et al., 2011), a higher degree and faster rate of neurodegeneration (Moffat et al., 2000; Caroli and Frisoni, 2010), alterations in brain function and glucose metabolism (Bookheimer et al., 2000; Bondi et al., 2005; Langbaum et al., 2009), changes in cerebrospinal fluid (CSF) measures of amyloid and tau (Vemuri et al., 2010; Tosun et al., 2011), as well as more impaired cognition (Mayeux et al., 2001; Farlow et al., 2004; Caselli et al., 2011) in patients with L-MCI and AD and cognitively healthy older adults (HC). However, the role of *APOE* genotype in E-MCI has not been assessed. Therefore, the goal of this study is to evaluate the effect of *APOE* ε4 status on amyloid deposition, neurodegeneration, and cognition in patients diagnosed with E-MCI, the earliest clinically-defined prodromal stage of AD.

## Materials and methods

### Alzheimer's disease neuroimaging initiative (ADNI)

ADNI was launched in 2004 by the National Institute on Aging (NIA), the National Institute of Biomedical Imaging and Bioengineering (NIBIB), the Food and Drug Administration (FDA), pharmaceutical companies, and non-profit organizations, as a multi-year public-private partnership. The Principal Investigator is Michael W. Weiner, MD, VA Medical Center and UCSF. ADNI is a longitudinal study, ultimately including more than 1200 participants (aged 55–90) recruited from over 50 sites across the United States and Canada. Further information can be found at http://www.adni-info.org/ and in previous reports (Jack et al., 2010; Jagust et al., 2010; Petersen et al., 2010; Saykin et al., 2010; Trojanowski et al., 2010; Weiner et al., 2010). Appropriate Institutional Review Boards approval occurred at each ADNI site and informed consent was obtained from each participant or authorized representative.

### Participants

Participants were selected if they were designated as E-MCI or HC (continuing participants or newly enrolled) at the initial visit of the ADNI-GO or ADNI-2 phases and had *APOE* genotype data. The sample included 209 E-MCI patients and 123 HC. Patients were diagnosed with E-MCI using criteria described in the ADNI-2 procedures manual (http://www.adni-info.org/). Briefly, patients were diagnosed with E-MCI using the following criteria:

1. Subject must have a subjective memory concern as reported by subject, study partner, or clinician.
2. Abnormal memory function documented by scoring within the education adjusted ranges on the Logical Memory II subscale (Delayed Paragraph Recall, Paragraph A only) from the Wechsler Memory Scale—Revised (the maximum score is 25):
  a. 9–11 for 16 or more years of education.
  b. 5–9 for 8–15 years of education.
  c. 3–6 for 0–7 years of education.
3. Mini-Mental State Exam score between 24 and 30 (inclusive) (Exceptions may be made for subjects with less than 8 years of education at the discretion of the project director).
4. Clinical Dementia Rating = 0.5; Memory Box score must be at least 0.5.
5. General cognition and functional performance sufficiently preserved such that a diagnosis of Alzheimer's disease cannot be made by the site physician at the time of the screening visit.

In addition, all participants met ADNI inclusion and exclusion criteria which have been described previously (Weiner et al., 2010) and can be found at http://www.adni-info.org/.

*APOE* genotyping for all participants was performed as previously described (Saykin et al., 2010). In the present study, we sought to evaluate the impact of the presence or absence of an *APOE* ε4 allele on imaging and non-imaging phenotypes. Therefore, all participants were divided into two groups based on *APOE* ε4 status, including participants with one or more ε4 allele (*APOE* ε4 positive (ε4+); 85 E-MCI, 30 HC) and participants without an ε4 allele (*APOE* ε4 negative (ε4−); 124 E-MCI, 93 HC).

### Clinical and neuropsychological assessments

All clinical and neuropsychological test performance data for included participants was downloaded from the ADNI clinical data repository on the Laboratory of Neuro Imaging (LONI) site. Specifically, we evaluated participant performance on the Mini-Mental State Exam (MMSE), Alzheimer's Disease Assessment Scale (ADAS), Montreal Cognitive Assessment (MoCA; Total and all sub-scores), Rey Auditory Verbal Learning Test (RAVLT; Total score, delayed recall score, delayed recognition score), Weschler's Logical Memory Scale—Revised (LM; Immediate and Delayed), Clock Drawing Test (CDT), Trailmaking Test A and B (TMT-A, TMT-B), Boston Naming Test (BNT), Animal Fluency, and the American National Adult Reading Test (ANART). We also evaluated clinical measures, including a measure of dementia severity [Clinical Dementia Rating Scale (CDR), Sum of Boxes score], general functioning [Functional Assessment Questionnaire (FAQ)], depression [Geriatric Depression Scale (GDS)], and stroke/vascular incident history (Modified Hachinski Scale). Cognitive complaints were assessed using the Measure of Everyday Cognition (E-Cog) from both the patient and an informant. The total level of complaints on the E-Cog (overall and within each domain) for both the participant and the informant were assessed as percentage of items endorsed as either “2 = questionably or occasionally worse,” “3 = consistently a little worse,” or “4 = consistently much worse.” Items endorsed as “9 = I don't know” were excluded.

### Structural MRI scans

All available baseline 3 Tesla structural magnetic resonance imaging (MRI) scans were downloaded from LONI for included E-MCI and HC participants. Scans were corrected prior to download as previously described (Jack et al., 2008, 2010). Most participants had a minimum of two scans from the baseline visit. All available scans were processed using voxel-based morphometry (VBM) implemented in Statistical Parametric Mapping 8 (SPM8) (Ashburner and Friston, 2000) and Freesurfer version 5.1 (Dale et al., 1999; Fischl et al., 1999), as described in previous reports (Dale et al., 1999; Fischl et al., 1999; Ashburner and Friston, 2000; Risacher et al., 2009, 2010) and briefly below:

#### VBM

Scans were co-registered to a T1-weighted template, segmented into grey matter (GM), white matter (WM), and CSF compartments with bias correction, unmodulated normalized to Montreal Neurologic Institute (MNI) space as 1 × 1 × 1 mm voxels, and smoothed with an 8 mm Gaussian kernel. All scans underwent extensive quality control. Mean GM density was extracted from all available baseline scans for target regions of interest (ROIs) using MarsBaR (Brett et al., 2002). Since most participants had two or more baseline MRI scans, an average GM density measure was calculated for each ROI using the mean GM density values extracted from each of the available baseline scans. Eighteen participants (5 HC ε4−, 2 HC ε4+, 5 E-MCI ε4−, 6 E-MCI ε4+) were excluded from the GM density analyses for missing data or failed processing.

#### Automated parcellation

Freesurfer version 5.1 was used to extract volumetric and cortical thickness measures. Similar to the VBM ROI data, values from all available baseline scans were averaged to create a mean volumetric or cortical thickness value for each ROI. Seven participants (2 HC ε4−, 1 HC ε4+, 2 E-MCI ε4−, 2 E-MCI ε4+) were excluded from the cortical thickness and volumetric analyses for incomplete data or failed processing.

### Amyloid pet scans ([^11^C]florbetapir)

Pre-processed [^11^C]Florbetapir PET scans (Coregistered, Averaged, Standardized Image and Voxel Size, Uniform Resolution) were downloaded from LONI (http://adni.loni.ucla.edu/). Before download, images were averaged, aligned to a standard space, re-sampled to a standard image and voxel size, smoothed to a uniform resolution and normalized to a cerebellar GM reference region resulting in standardized uptake value ratio (SUVR) images as previously described (Jagust et al., 2010). After downloading, the images were aligned to each participant's same visit MRI scan and normalized to MNI space as 2 × 2 × 2 mm voxels using parameters from the MRI segmentation. The normalized scans were evaluated for the effect of *APOE* ε4 status on a voxel-wise basis using a two-sample *t*-test, masked using a whole-brain mask, and covaried for age, gender, education, and handedness. Significant results were displayed at a voxel-wise threshold of *p* < 0.01 [family-wise error (FWE) correction for multiple comparisons] with a minimum cluster size (k) of 50 voxels. SPM8 was used for all processing and voxel-wise analysis. Mean regional SUVR values were also extracted for target ROIs using MarsBaR. Fourteen participants (6 HC ε4−, 5 HC ε4+, 3 E-MCI ε4−) were excluded from [^11^C]Florbetapir analyses for missing scan data or failed processing.

### CSF biomarkers

Levels of amyloid-beta 1-42 (Aβ), total tau, and phosphorylated tau (p-tau) were measured from all available CSF samples as previously described (Shaw et al., 2009, 2011; Trojanowski et al., 2010). CSF data was downloaded from the LONI site and extracted for all included participants. Of the 332 included participants, 44 participants (25 E-MCI and 19 HC) were missing all CSF data. 4 additional participants (2 E-MCI, 3 HC) were missing CSF tau data and 2 additional HC participants were missing CSF p-tau data. Furthermore, participants with CSF levels outside 3 SDs above or below the mean were excluded, including 6 E-MCI participants with tau levels more than 3 SDs above the mean and 2 E-MCI participants with p-tau levels more than 3 SDs above the mean. Thus, the final samples for CSF analyses included 288 participants in the CSF Aβ analysis, 278 participants in the CSF tau analysis, and 284 participants in the CSF p-tau analysis.

### Statistical analyses

We evaluated the effect of diagnosis and *APOE* ε4 status on demographics, cognition, cognitive complaints, amyloid deposition, atrophy, and CSF biomarkers using two-way analysis of covariance (ANCOVA) for continuous variables and a chi-square test for categorical variables implemented in SPSS 19.0 (SPSS, Inc., Chicago, IL). Specifically, the effect of diagnosis (HC vs. E-MCI), *APOE* ε4 status (ε4+ vs. ε4−), and the interaction of diagnosis and ε4 status on performance on clinical and psychometric tests, cognitive complaints, amyloid deposition (mean SUVR from target ROIs), CSF levels of Aβ, tau, and p-tau, and brain atrophy (volume, cortical thickness, and GM density from target ROIs) were assessed. All ANCOVA analyses were covaried for age, gender, education, and handedness. The analysis of neurodegenerative measures was also covaried for total intracranial volume (ICV). The frequency of having one or more *APOE* ε4 alleles was also compared between diagnostic groups (HC vs. E-MCI) using a chi-square test.

## Results

### Demographics, psychometric performance, and cognitive complaints

Significantly more E-MCI were *APOE* ε4+ than HC (*p* = 0.003), with 85 of 209 E-MCI participants (40.7%) showing one or more ε4 alleles relative to only 30 of 123 HC participants (24.4%). Demographics and psychometric performance variables for E-MCI and HC participants stratified by *APOE* ε4 status are shown in Table 1. The effect of diagnosis, ε4 status, and the interaction between diagnosis and ε4 status are displayed. Age was significantly different between diagnostic groups (*p* < 0.05) but not *APOE* ε4 groups. A significant interaction between diagnosis and ε4 status on LM Immediate and Delayed performance was observed, with ε4+ HC showing worse performance on both measures than ε4− HC participants but no difference by ε4 status in E-MCI participants. A trend for a significant interaction on the MoCA delayed recall sub-score (*p* = 0.05) was also observed, again with a significant effect of ε4 status in HC but not E-MCI participants. Finally, a marginally significant interaction of diagnosis and ε4 status for informant complaints in the visuospatial domain (*p* = 0.05) was also seen, with ε4 status having an effect only in E-MCI participants.

**Table 1:** **Demographics, neuropsychological and clinical test performance, and cognitive complaints [Mean (Standard Error)]**.

		**HC:** ε4− (*n* = 93)	**HC:** ε4+ (*n* = 30)	**E-MCI:** ε4− (*n* = 124)	**E-MCI:** ε4+ (*n* = 85)	***p*-values**		
						**DX**	***APOE***	**Interaction**
Demographics	Age (years)	74.1, (0.72)	73.67, (1.27)	71.47, (0.62)	70.26, (0.75)	0.0007	0.3506	0.6572
	Education (years)	16.47, (0.27)	16.37, (0.48)	15.9, (0.24)	15.78, (0.28)	0.0810	0.7251	0.9756
	Gender (M, F)	49, 44	13, 17	64, 60	52, 33	0.3686	0.4394	0.3258
	Handedness (R, L)	85, 8	28, 2	109, 15	79, 6	0.5619	0.2777	0.5831
Clinical performance	CDR-Sum of Boxes	0.04, (0.06)	0.08, (0.11)	1.15, (0.05)	1.34, (0.06)	0.0000	0.0979	0.2989
	FAQ Total^a^	0.17, (0.25)	0.05, (0.45)	1.66, (0.21)	2.22, (0.26)	0.0000	0.4699	0.2637
	Modified Hachinski Total	0.59, (0.07)	0.37, (0.12)	0.77, (0.06)	0.58, (0.07)	0.0257	0.0164	0.8585
	GDS Total^b^	0.80, (0.14)	0.53, (0.25)	1.87, (0.12)	1.55, (0.15)	0.0000	0.0901	0.8687
Reading	ANART Errors^c^	10.61, (0.83)	9.05, (1.47)	11.22, (0.71)	12.35, (0.88)	0.0606	0.8320	0.1832
General cognition	MMSE Total Score	29.05, (0.14)	28.91, (0.25)	28.57, (0.12)	28.06, (0.15)	0.0002	0.0565	0.2847
	ADAS Cognitive Subtotal^d^	6.25, (0.36)	6.76, (0.63)	7.77, (0.30)	8.60, (0.37)	0.0002	0.1211	0.7147
	ADAS Total Score^e^	9.63, (0.51)	10.38, (0.90)	11.99, (0.44)	13.98, (0.53)	0.0000	0.0266	0.3168
	MoCA Total Score^f^	25.64, (0.27)	24.90, (0.47)	24.12, (0.22)	23.57, (0.28)	0.0000	0.0446	0.7835
Memory	Logical Memory - Immediate	14.78, (0.29)	13.26, (0.50)	10.84, (0.24)	10.85, (0.30)	0.0000	0.0273	0.0267
	Logical Memory - Delayed	13.89, (0.23)	12.51, (0.39)	8.76, (0.19)	8.97, (0.24)	0.0000	0.0318	0.0036
	RAVLT Total Score^d^	44.99, (0.93)	42.48, (1.65)	39.98, (0.79)	37.63, (0.97)	0.0000	0.0320	0.9459
	RAVLT Delayed Recall^d^	7.26, (0.39)	6.61, (0.70)	6.18, (0.33)	5.24, (0.41)	0.0128	0.0976	0.7601
	RAVLT Delayed Recognition^d^	12.82, (0.27)	12.20, (0.48)	11.98, (0.23)	12.01, (0.28)	0.1249	0.3687	0.3238
	MoCA Delayed Memory^g^	2.36, (0.16)	1.67, (0.29)	1.40, (0.14)	1.49, (0.17)	0.0061	0.1356	0.0509
Language and naming	Boston Naming Test Total^e^	28.24, (0.31)	28.64, (0.55)	27.08, (0.26)	27.34, (0.32)	0.0015	0.3790	0.8413
	Animal Fluency Total^d^	21.07, (0.49)	21.63, (0.87)	18.98, (0.42)	18.62, (0.51)	0.0000	0.8704	0.4444
	MoCA Naming^d^	2.90, (0.03)	3.01, (0.06)	2.83, (0.03)	2.89, (0.04)	0.0311	0.0374	0.5251
	MoCA Language^d^	2.52, (0.08)	2.57, (0.13)	2.45, (0.06)	2.34, (0.08)	0.1150	0.7619	0.3858
Visuospatial and executive function	Clock Drawing Score^d^	4.62, (0.06)	4.63, (0.11)	4.69, (0.05)	4.60, (0.07)	0.8319	0.5891	0.4899
	Clock Drawing - Copy Score^d^	4.86, (0.04)	4.93, (0.08)	4.80, (0.04)	4.84, (0.05)	0.2157	0.3119	0.7972
	Trailmaking A^d^	33.40, (1.16)	34.28, (2.05)	34.52, (0.99)	39.06, (1.21)	0.0418	0.0542	0.1943
	Trailmaking B^h^	81.07, (4.34)	79.47, (7.85)	90.52, (3.76)	107.64, (4.58)	0.0007	0.1469	0.0807
	Trailmaking B-A^h^	47.66, (3.99)	45.54, (7.21)	55.94, (3.45)	68.52, (4.2)	0.0021	0.2868	0.1347
	MoCA Visuospatial-Executive^a^	4.29, (0.09)	4.37, (0.16)	4.28, (0.08)	4.04, (0.10)	0.1440	0.4833	0.1444
	MoCA Attention^d^	5.77, (0.07)	5.55, (0.12)	5.64, (0.06)	5.47, (0.07)	0.2040	0.0123	0.7272
	MoCA Abstraction^d^	1.80, (0.06)	1.79, (0.11)	1.69, (0.05)	1.56, (0.06)	0.0276	0.3399	0.4321
	MoCA Orientation^d^	5.94, (0.04)	5.94, (0.08)	5.83, (0.04)	5.74, (0.04)	0.0040	0.3920	0.4418
Patient complaints	E-Cog Patient: Total^d^^,^^k^	27.01, (2.59)	28.00, (4.52)	49.04, (2.18)	52.10, (2.67)	0.0000	0.5147	0.7396
	E-Cog Patient: Memory^d^^,^^k^	43.09, (2.92)	45.91, (5.11)	73.03, (2.46)	77.60, (3.02)	0.0000	0.2932	0.8033
	E-Cog Patient: Language^d^^,^^k^	32.59, (3.16)	33.39, (5.52)	56.31, (2.66)	55.96, (3.26)	0.0000	0.9520	0.8805
	E-Cog Patient: Visuospatial^d^^,^^k^	11.55, (3.11)	13.91, (5.43)	28.44, (2.61)	33.46, (3.20)	0.0000	0.3222	0.7216
	E-Cog Patient: Planning^d^^,^^k^	12.94, (3.33)	11.29, (5.82)	32.82, (2.80)	38.40, (3.43)	0.0000	0.6230	0.3654
	E-Cog Patient: Organization^d^^,^^k^	20.69, (3.56)	17.07, (6.22)	38.33, (2.99)	38.16, (3.67)	0.0000	0.6576	0.6858
	E-Cog Patient: Divided Attention^d^^,^^k^	36.55, (4.10)	41.95, (7.16)	59.36, (3.44)	64.13, (4.23)	0.0000	0.3011	0.9485
Informant complaints	E-Cog Informant: Total^i^^,^^k^	11.38, (2.39)	14.83, (4.18)	40.00, (2.01)	45.78, (2.47)	0.0000	0.1087	0.6866
	E-Cog Informant: Memory^i^^,^^k^	21.04, (2.98)	25.77, (5.21)	61.73, (2.51)	64.77, (3.08)	0.0000	0.2778	0.8138
	E-Cog Informant: Language^i^^,^^k^	8.71, (2.95)	15.11, (5.16)	38.32, (2.48)	44.24, (3.05)	0.0000	0.0829	0.9454
	E-Cog Informant: Visuospatial^j^^,^^k^	5.42, (2.78)	4.18, (4.86)	21.23, (2.35)	32.97, (2.87)	0.0000	0.1166	0.0528
	E-Cog Informant: Planning^i^^,^^k^	7.44, (3.25)	10.97, (5.68)	31.80, (2.73)	37.94, (3.35)	0.0000	0.2155	0.7375
	E-Cog Informant: Organization^i^^,^^k^	7.62, (3.25)	9.34, (5.68)	32.36, (2.73)	37.20, (3.35)	0.0000	0.4009	0.6891
	E-Cog Informant: Divided Attention^i^^,^^k^	18.38, (4.07)	23.69, (7.12)	56.69, (3.43)	57.89, (4.20)	0.0000	0.5051	0.6736

a *2 HC (1 ε4+, 1 ε4−) missing data*.

b *2 E-MCI (1 ε4+, 1 ε4−) missing data*.

c *2 HC (1 ε4+, 1 ε4−), 5 E-MCI (2 ε4+, 3 ε4−) missing data*.

d *1 HC (ε4+) missing data*.

e *1 HC (ε4+), 1 E-MCI (ε4−) missing data*.

f *3 HC (1 ε4+, 2 ε4−), 1 E-MCI (ε4+) missing data*.

g *2 HC (1 ε4+, 1 ε4−), 1 E-MCI (ε4+) missing data*.

h *2 HC (2 ε4+), 4 E-MCI (1 ε4+, 3 ε4−) missing data*.

i *4 HC (1 ε4+, 3 ε4−) missing data*.

j *4 HC (ε4+), 1 E-MCI (ε4−) missing data*.

k *Patient and informant E-Cog values are expressed as a percentage of items endorsed (total and within each domain); see text for additional description*.

Significant effects of diagnosis on the CDR-SB, FAQ, Modified Hachinski Total, and GDS were observed (*p* < 0.05), with E-MCI participants showing a greater CDR-SB, as well as higher scores on the FAQ, Modified Hachinski, and GDS. Differences in psychometric performance by diagnosis were observed for nearly every test (*p* < 0.05), except for the RAVLT Delayed Recognition, CDT (Total and Copy Scores), and the MoCA language, executive-visuospatial function, and attention sub-scores. Significant differences in cognitive complaints from both the participant and the informant by diagnosis were also observed in all domains (*p* < 0.001). In all cases, E-MCI participants had worse cognition and more cognitive complaints than HC participants.

Vascular risk factors and/or stroke history was significantly different by *APOE* ε4 status (*p* < 0.05), with ε4+ participants showing lower Modified Hachinski Total scores. In addition, ε4 status was significantly associated with performance on a number of psychometric tests, including the ADAS Total score, MoCA Total score, RAVLT Total score, and the MoCA naming and attention sub-scores (*p* < 0.05). The effect of ε4 status was also significant at a trend level for TMT-A (*p* = 0.05). For these comparisons, ε4+ participants demonstrated worse performance than ε4−.

### Voxel-based comparisons of amyloid deposition

**Figure 1:**
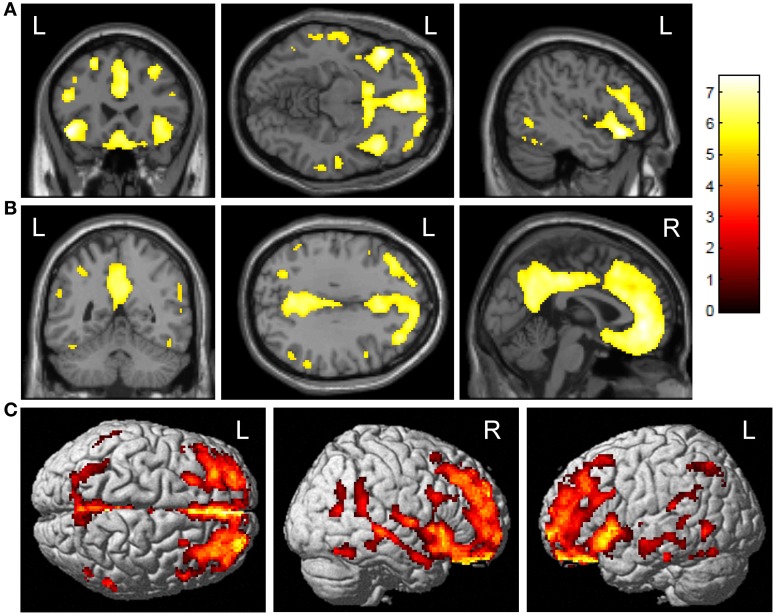
**Voxel-wise association of *APOE* ε4 status and amyloid deposition in E-MCI participants.** Greater cortical amyloid deposition was observed in *APOE* ε4+ (*n* = 85) relative to *APOE* ε4− (*n* = 121) E-MCI participants. Significant clusters were observed in the medial and lateral frontal lobes **(A)**, anterior and posterior cingulate **(B)**, and lateral temporal lobes. Surface renderings show the diffuse pattern of significant clusters **(C)**. All analyses were covaried for age, gender, education, and handedness and a voxel-wise threshold of *p* < 0.01 (FWE correction for multiple comparisons) and minimum cluster size (*k*) of 50 voxels was considered significant.

ε4+ E-MCI showed significantly greater amyloid deposition upon voxel-wise analysis than ε4− (Figure 1; voxel-wise threshold: *p* < 0.01 (FWE), *k* = 50 voxels). The most significant cluster was observed in the left orbitofrontal cortex (Figure 1A). Additional significant clusters were observed in the medial frontal lobe/anterior cingulate cortex, the right orbitofrontal cortex, and the posterior cingulate/precuneus (Figure 1B). The surface rendering also reflects the widespread pattern of significant differences with significant clusters throughout the frontal, parietal, and temporal lobes (Figure 1C). No significant clusters were observed in the reverse comparison (ε4− > ε4+; *data not shown*).

### ROI comparisons of amyloid deposition

**Figure 2:**
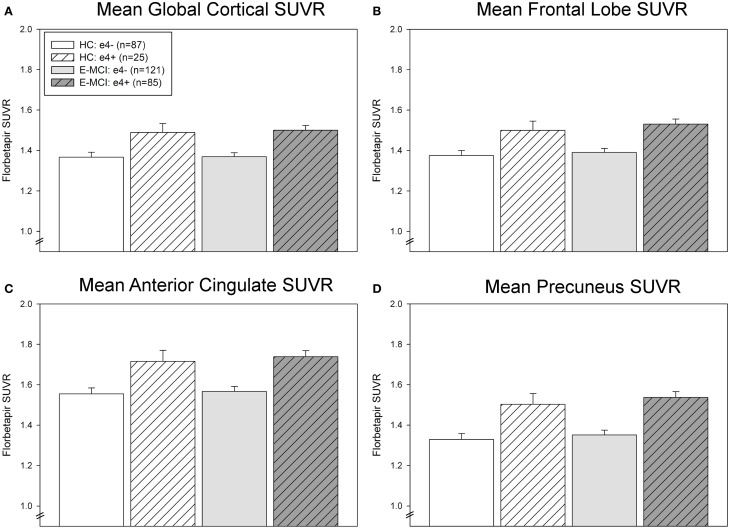
**Regional effects of *APOE* ε4 status on amyloid deposition in E-MCI and HC participants.** A significant effect of *APOE* ε4 status on regional amyloid deposition was observed (*p* < 0.001), including in the global cortical mean amyloid **(A)**, mean bilateral frontal lobes **(B)**, anterior cingulate **(C)**, and precuneus **(D)**. In all evaluated regions, *APOE* ε4+ E-MCI (*n* = 85) and HC (*n* = 25) participants showed a higher mean standardized uptake value ratio (SUVR) than *APOE* ε4− E-MCI (*n* = 121) and HC (*n* = 87) participants. No significant effect of diagnostic status was observed.

ROI results were consistent with voxel-wise findings demonstrating significantly greater global and regional amyloid deposition in ε4+ relative to ε4− E-MCI participants in the global cortex, mean frontal lobe, anterior cingulate, and precuneus (Figure 2). A significant effect of ε4 status (*p* < 0.001) but not diagnosis was observed in all ROI measures, with ε4+ participants showing greater amyloid than ε4− participants regardless of diagnosis (HC or E-MCI). Overall, amyloid PET results indicate that ε4+ individuals showed greater amyloid deposition than ε4− regardless of cognitive impairment in the earliest stages of decline.

### ROI comparisons of neurodegeneration

**Figure 3:**
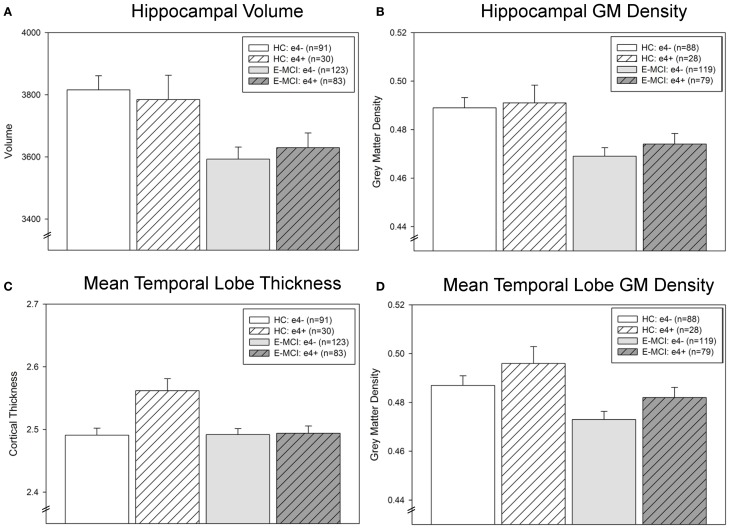
**Temporal lobe atrophy is associated with *APOE* ε4 status and diagnostic group in E-MCI and HC participants.** A significant effect of diagnosis on neurodegeneration in the temporal lobe was observed (*p* < 0.05), including in hippocampal volume **(A)** and grey matter (GM) density **(B)**, mean temporal lobe cortical thickness **(C)**, and mean temporal lobe GM density **(D)**. In all evaluated regions, E-MCI participants showed more temporal lobe atrophy than HC participants. In addition, *APOE* ε4 status was significantly associated with mean temporal lobe GM density, with *APOE* ε4− participants showing smaller mean temporal lobe GM density than *APOE* ε4+ participants. Finally, a significant interaction effect of diagnosis and *APOE* ε4 status was observed in mean temporal lobe cortical thickness, with ε4− HC participants showing thicker mean temporal lobes than all other groups. All analyses were covaried for age, gender, education, handedness, and total intracranial volume (ICV). The total number of participants for each analysis is indicated on each graph (Panels **A–D**). Note: Thirteen participants were excluded from the GM density analyses, but not the cortical thickness and volumetric analyses, for failed VBM segmentation (3 HC ε4−, 2 HC ε4+, 4 E-MCI ε4−, 4 E-MCI ε4+).

Hippocampal neurodegeneration (volume and GM density) was associated with diagnosis (*p* < 0.001; Figures 3A,B) but not *APOE* ε4 status. E-MCI participants showed more hippocampal atrophy than HC. However, a significant interaction effect of diagnosis and ε4 status on mean temporal lobe cortical thickness was observed (*p* = 0.008; Figure 3C), with ε4+ HC participants showing thicker mean temporal lobes than all other groups. Mean temporal lobe GM density was also significantly associated with diagnosis (*p* = 0.005) and ε4 status (*p* = 0.047; Figure 3D), as E-MCI patients showed smaller mean temporal lobe GM density than HC and ε4− participants showed smaller mean temporal lobe GM density than ε4+ participants.

### CSF levels of Aβ, tau, and p-tau

**Figure 4:**
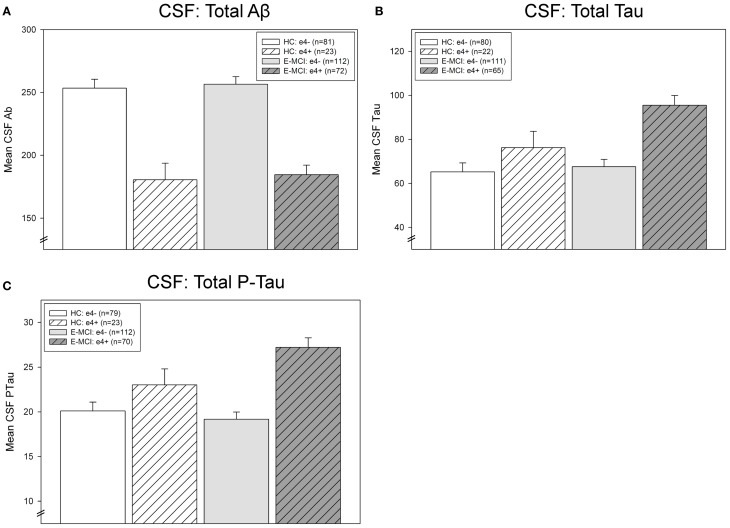
**Effects of *APOE* ε4 status and diagnosis on CSF protein levels in E-MCI and HC participants.** Diagnostic group and *APOE* ε4 status significantly affected CSF levels of Aβ **(A)**, total tau **(B)**, and p-tau **(C)**. CSF Aβ was significantly associated to *APOE* ε4 status (*p* < 0.001), with *APOE* ε4+ E-MCI and HC participants showing lower levels of Aβ than *APOE* ε4− E-MCI and HC participants, regardless of diagnosis. Total tau levels were significantly affected by both diagnosis (*p* < 0.05) and *APOE* ε4 status (*p* < 0.001), with E-MCI patients showing higher total tau levels than HC participants and *APOE* ε4+ showing higher levels than *APOE* ε4− participants. Finally, an interaction between diagnostic status and *APOE* ε4 status on CSF p-tau levels was also observed (*p* < 0.05), primarily driven by a higher level of p-tau in *APOE* ε4+ HC and E-MCI participants relative to *APOE* ε4− HC and E-MCI participants. The total number of participants in each analysis is shown for each graph (Panels **A–C**). Note: See text for description of participants excluded from the CSF analysis.

CSF levels of Aβ, tau, and p-tau were significantly affected by diagnosis and *APOE* ε4 status (Figure 4). Levels of CSF Aβ were significantly associated with ε4 status (*p* < 0.001), with ε4+ participants showing lower levels of Aβ than ε4− participants (Figure 4A). CSF tau levels were significantly affected by both diagnosis (*p* = 0.041) and ε4 status (*p* < 0.001; Figure 4B). E-MCI patients had higher tau levels than HC participants and ε4+ participants had higher levels than ε4− participants. Finally, an interaction between diagnosis and ε4 status on p-tau was also observed (*p* = 0.046), primarily driven by a higher level of p-tau in ε4+ HC and E-MCI participants (Figure 4C).

## Discussion

This study provides a comprehensive evaluation of the impact of *APOE* ε4 status on cognition, cognitive complaints, amyloid deposition, neurodegeneration, and CSF Aβ, tau, and p-tau levels in E-MCI and HC. As expected, we observed a significant association of diagnosis with clinical and cognitive status. Furthermore, diagnosis was associated with neurodegeneration and CSF tau and p-tau levels but not with amyloid deposition. Cognitive performance, amyloid deposition, temporal lobe atrophy, and CSF tau and p-tau levels were significantly associated with ε4 status, with ε4+ participants showing poorer cognition, less temporal lobe atrophy, and higher CSF tau and p-tau levels. ε4+ participants also showed greater cortical amyloid deposition and lower CSF Aβ levels. Finally, an interaction between diagnosis and ε4 status was observed for memory performance, temporal lobe cortical thickness, and CSF p-tau levels. Overall, the results suggest that *APOE* ε4 status impacts AD-related pathological and clinical changes in E-MCI and HC.

The effect of *APOE* genotype on amyloid deposition has been shown previously, including in middle-aged and older cognitively healthy adults, as well as patients with L-MCI and AD (Drzezga et al., 2009; Shaw et al., 2009; Morris et al., 2010; Fleisher et al., 2011; Tosun et al., 2011). Biochemically, *APOE* genotype has been shown to affect Aβ clearance rate, with the APOE ε4 isoform showing significantly slower clearance (Deane et al., 2008; Castellano et al., 2011; Holtzman et al., 2012). The lack of diagnostic effect on amyloid deposition in this study suggests that in the earliest stages of cognitive change, *APOE* ε4 status has a stronger relationship to amyloid deposition than cognitive status.

The additional findings of a diagnostic effect on cognition, cognitive complaints, neurodegeneration, and CSF tau and p-tau levels underscore the importance of E-MCI as a diagnostic entity. Thus, this report has notable clinical implications, particularly in the potential implementation and utilization of E-MCI as a clinical diagnostic entity. Patients with E-MCI show changes in cognition and selected biomarkers, suggesting that these individuals may have a higher likelihood of clinical progression. The association of cognition and complaints to atrophic changes, rather than amyloid levels, supports E-MCI as an intermediate stage with pathology beyond amyloid accumulation.

These results further support the Jack et al. model of AD biomarkers, suggesting that changes in cognition and neurodegeneration occur after measurable amyloid accumulation (Saykin et al., 2010; Jack et al., 2011). Additionally, *APOE* ε4 genotype may alter the hypothesized sigmoidal curves, in particular amyloid accumulation. These results also indicate the importance of genetic background in determining likelihood and extent of amyloid accumulation, even in preclinical stages, which may be particularly important in clinical trial enrollment. Further, in the era of personalized medicine, the implications of *APOE* genotype disclosure to patients in a clinical setting must be carefully considered, given the impact of *APOE* on AD risk and amyloid deposition (Green et al., 2009; Roberts et al., 2011).

The observed greater temporal lobe cortical thickness and GM density in ε4+ participants, particularly in HC, is somewhat unexpected and may be related to the modest sample size of the ε4+ HC group. However, previous studies have observed increased cortical thickness, including in middle-aged *APOE* ε4 positive participants (Espeseth et al., 2008), in cognitively HC who are transitioning to become CSF Aβ biomarker positive (Fortea et al., 2011), and in asymptomatic patients positive for a *PSEN1* mutation more than 9 years prior to the clinical onset (Fortea et al., 2010). Thus, future studies including longitudinal follow-up with an expanded sample will be important in determining the significance of this finding.

The present study has a few notable limitations. First, we evaluated the effect of *APOE* ε4 status on AD biomarkers in only HC and E-MCI rather than across the disease spectrum. Although our goal was to evaluate *APOE* in the earliest stages of AD, future studies assessing the full clinical spectrum are warranted. In addition, we did not evaluate all known biomarkers of AD, including FDG PET or advanced MRI techniques (i.e., diffusion tensor imaging, resting-state functional MRI, etc.). These measures are available in subsets of the ADNI-GO/2 cohort and thus, future studies evaluating these measures would augment the findings of the present report. Thirdly, genome-wide genetic data for this cohort was recently released. Future studies assessing other variants may provide information about the role of genetics in very early stages of AD. Finally, the present study evaluates only cross-sectional measures. Future studies using longitudinal and clinical outcome data will allow assessment of the role of *APOE* in progression of HC and E-MCI.

In summary, we assessed the role of *APOE* ε4 status on clinical and cognitive measures, cognitive complaints, and imaging and CSF biomarkers in HC and E-MCI participants from the ADNI-GO/2 cohort. We determined that *APOE* ε4 status is associated with increased amyloid deposition in both HC and E-MCI, while diagnostic category is associated with measures of cognition and cognitive complaints, as well as neurodegeneration. Therefore, we conclude that *APOE* is an important mediator of amyloid pathology in the earliest stages of AD-associated clinical decline.

### Conflict of interest statement

Dr. Jack serves as a consultant for Janssen, Bristol-Meyer-Squibb, General Electric, Siements, and Johnson & Johnson; is involved in clinical trials sponsored by Allon and Baxter, Inc.; receives research funding from the NIH and the Alexander Family Alzheimer's Disease Research Professorship of the Mayo Foundation. Dr. Aisen serves on a scientific advisory board for NeuroPhage; serves as a consultant to Elan Corporation, Wyeth, Eisai Inc., Schering-Plough Corp., Bristol-Myers-Squibb, Eli Lilly and Company, NeuroPhage, Merck & Co., Roche, Amgen, Genentech, Inc., Abbott, Pfizer Inc., Novartis, Bayer, Astellas, Dainippon, Biomarin, Solvay, Otsuka, Daiichi, AstraZeneca, Janssen and Medivation, Inc.; and receives research support from Pfizer Inc., Eli Lilly and Company, and Baxter International Inc. Dr. Jagust has served on a scientific advisory board for Genentech, Inc.; has served as a consultant for Synarc, Janssen Alzheimer Immunotherapy, Genentech, Inc., Siemens, and TauRx. Dr. Shaw has served on a technical advisory board for Innogenetics and Saladax; serves as a consultant for Janssen; is involved in clinical trials sponsored by Eisai, Inc., and Baxter, Inc.; and receives research funding from the NIH and MJ Fox Foundation for Parkinson's research. Dr. Trojanowski may accrue revenue in the future on patents submitted by the University of Pennsylvania wherein he is co-Inventor and he received revenue from the sale of Avid to Eli Lily as co-inventor on imaging related patents submitted by the University of Pennsylvania; and receives research support from the NIH, Bristol Myer Squib, AstraZenica and several non-profits. Dr. Weiner has served on scientific advisory boards for Eli Lilly, Araclon, Institut Catala de Neurociencies Aplicades, the Gulf War Veterans Illnesses Advisory Committee, Biogen Idec, and Pfizer; has served as a consultant to Astra Zeneca, Araclon, Medivation/Pfizer, Ipsen, TauRx Therapeutics LDT, Bayer Healthcare, Biogen Idec, Exonhit Therapeutics, Servier, Synarc, Janssen, Harvard University, and KLJ Associates; has received funding for travel from NeuroVigil, Inc., CHRU Hopital Roger Salengro, Siemens, AstraZeneca, Geneva University Hospitals, Eli Lilly, Paris University, Institut Catala de Neurociencies Aplicades, University of New Mexico School of Medicine, Ipsen, Clinical Trials on Alzheimer's Disease, the AD/PD Meeting, Paul Sabatier University, Novartis, Tohoku University, Fundacio ACE, and Travel eDreams, Inc.; has received honoraria from NeuroVigil, Inc., Institut Catala de Neurociencies Aplicades, PMDA/Japanese Ministry of Health, Labour, and Welfare, Tohoku University, and the Alzheimer's Drug Discovery Foundation; has received research support from Merck and Avid; and has stock options for Synarc and Elan. Dr. Saykin has received investigator-initiated research funding from Welch Allyn and Siemens Healthcare; has served as a consultant or advisory board member for Siemens Healthcare and Eli Lilly. Drs. Risacher, Shen, Kim, Nho, Foroud, Petersen, and Koeppe declare that the research was conducted in the absence of any commercial or financial relationships that could be construed as a potential conflict of interest.
